# Artificial intelligence for diagnosing rare bone diseases: a global survey of healthcare professionals

**DOI:** 10.1186/s13023-025-03875-1

**Published:** 2025-07-16

**Authors:** Behnam Javanmardi, Rebekah L. Waikel, Tinatin Tkemaladze, Shahida Moosa, Alexander Küsshauer, Jean Tori Pantel, Minu Fardipour, Peter Krawitz, Benjamin D. Solomon, Klaus Mohnike

**Affiliations:** 1 Institute for Genomic Statistics and Bioinformatics, University Hospital Bonn, Bonn, Germany; 2 National Human Genome Research Institute, Bethesda, MD USA; 3 Department of Molecular and Medical Genetics, Tbilisi State Medical University, Tbilisi, Georgia; 4 Division of Clinical Genetics, Givi Zhvania Pediatric University Clinic, Tbilisi State Medical University, Tbilisi, Georgia; 5 Division of Molecular Biology and Human Genetics, Faculty of Medicine and Health Sciences, Stellenbosch University, Cape Town, South Africa; 6 Transfer Center enaCom, University of Bonn, Bonn, Germany; 7 Institute for Digitalization and General Medicine, Medical Faculty, RWTH Aachen University, Aachen, Germany; 8 University Hospital Magdeburg, Magdeburg, Germany

## Abstract

**Background:**

Rare bone diseases (RBDs) are an important group of conditions characterized by abnormalities in bone and cartilage. Their large number, individual rarity, and heterogeneity make accurate and timely diagnosis challenging. Establishing correlations between genotype and phenotype (mainly via imaging) is critical for diagnosing RBDs. Image recognition artificial intelligence (AI) has the potential to significantly improve the diagnostic process by assisting healthcare providers to identify and differentiate imaging patterns associated with various RBDs. This survey study sought to assess the interest of various healthcare providers worldwide in utilizing an AI-based assistant tool for the differential diagnosis of RBDs.

**Method:**

Survey data were collected from March to September 2024. The survey was performed online and the link was disseminated via direct email, newsletters, and flyers at scientific talks and conferences.

**Results:**

We received 103 completed surveys, representing respondents from 27 different countries covering most global regions, but mostly from Europe, the United States, and Canada. The majority of the participants are physicians (n = 92, 89%) and primarily work at academic medical centers (n = 84, 81%). While each participant could select multiple specialties, the most frequent clinician types were medical geneticists, pediatricians, and endocrinologists, accounting for 71 (69%) of the respondents. Ninety-four (91%) of the respondents find imaging to be very or extremely important, and the majority (n = 84, 81%) consider X-rays to be the most important imaging modality. Although around half of the participants (n = 45) have concerns about AI-related errors and consider the explainability of AI algorithms to be very (42/103) or extremely (9/103) important, 81% of the respondents report that they are somewhat (n = 39) or extremely (n = 45) likely to consider integrating image recognition AI into their current diagnostic workflow.

**Conclusions:**

Most survey participants are open to integrating image recognition AI into their RBD diagnostic workflow. However, concerns about AI-related errors, privacy, and model interpretability highlight the importance of transparent collaboration between developers and healthcare professionals throughout the development process to ensure that such technologies are clinically trustworthy and practically adoptable.

**Supplementary Information:**

The online version contains supplementary material available at 10.1186/s13023-025-03875-1.

## Background

According to the most recent Nosology of Genetic Skeletal Disorders [1], there are over 700 known rare bone diseases (RBDs) that involve over 500 different genes. Though these diseases are individually rare, they collectively affect a large number of individuals. As with most rare diseases, diagnosing RBDs is inherently challenging, often requiring extensive time and multiple clinical visits, a process that can be frustrating for patients, and which on average takes ~ 5 years [2]. Effective treatment hinges on precisely identifying the type of RBD, emphasizing the importance of addressing the current diagnostic gap. Moreover, accurate diagnosis can have economic and societal impact, as available therapies are often highly specific and may involve highly variable access and costs [3].

The current diagnostic gap involves two main challenges: (1) due to the large number of RBDs, even expert clinicians may have encountered a limited number of affected individuals, and there are a limited number of experts. The first line of contact is usually general practitioners such as family doctors or general pediatricians, who are not trained in the diagnosis and management of RBDs [4]. This can significantly delay the identification and referral of these patients to experts (usually medical geneticists). (2) Although advances in DNA sequencing techniques have significantly improved the diagnosis of genetic conditions, being able to identify the cause of RBDs can be challenging due to incomplete understanding of genetic causes and other etiologic factors.

The past few years have involved an enormous expansion in the use of artificial intelligence (AI) methods in virtually all medical fields [5, 6]; this trend is anticipated to accelerate in the coming years. One relevant area is Next Generation Phenotyping (NGP), which in this context refers to the application of advanced computer vision techniques to medical imaging data of individuals with genetic conditions. There are already AI-based NGP tools that assist in the diagnosis of genetic diseases with characteristic patterns affecting the face (DeepGestalt [7] and GestaltMatcher [8]) as well as the eye (Eye2Gene [9]). These tools take images as input and provide a prioritized list of disorders (and/or disease-causing genes) that can help steer the differential diagnosis. At the heart of these technologies are advanced deep convolutional neural networks, which are trained using thousands of images from individuals with different rare disorders.

This same concept underlies the Bone2Gene AI, currently under development. Bone2Gene will be trained to identify and distinguish the unique imaging patterns linked to various RBDs. Initially, Bone2Gene AI will focus on training, using dorsopalmar radiographs of hands and wrists from various RBDs. This approach is selected because performing a hand X-ray for bone age assessment [10] is a common practice for children suspected of having bone irregularities. In subsequent stages, Bone2Gene will broaden its training to include radiographs from other areas of the body. As of this writing, the Bone2Gene dataset comprises approximately 2,700 hand X-rays from over 800 patients, covering more than 30 RBDs. The Bone2Gene team is currently developing multiple algorithms aiming at (i) detecting dysmorphic features in a hand X-ray, (ii) differentiating different patterns and outputting a list of most probable RBDs, and (iii) measuring different features of carpal, metacarpals, and phalangeal bones. The goal of all these algorithms is to provide the clinicians with relevant information for the differential diagnosis. See the Bone2Gene website (https://bone2gene.org) for current information.

While there is considerable current attention to AI, the attitudes and expectations of clinicians are critical to gauge prior to implementation. Following an approach inspired by the Lean Startup methodology [11, 12], which emphasizes the pivotal role of user feedback in shaping technological innovations, we aimed to gauge the interest of different healthcare providers across the world in having an AI-based assistant tool for the differential diagnosis of RBDs.

## Method

### The questionnaire

The survey included different groups of questions. First, we asked about participants’ demographics, specialties, the patient age groups they work with, and participant clinical experience with RBDs. Next, we collected data about the most frequent type of RBDs for which the participants provide care for. For that, we asked the participants to mark all the groups (according to the most recent revision of the nosology of RBDs [1]) that they or their healthcare facility have dealt with.

Further, we gauged the opinion of the participants on the importance of medical images (X-rays, MRI, etc.) and also the most important imaging modality for the postnatal diagnosis of RBDs. We also asked the participants how difficult they think it is to delineate between different RBDs based on visual inspection of patients’ radiographs (excluding the disorders with highly characteristic features such as achondroplasia).

In addition, we asked survey participants about their concerns regarding regulatory considerations, potential errors, and the importance of the explainability of AI. And finally, we gauged the perceived utility of AI-based diagnostic tools.

A list of the survey questions and the response options is provided in Table 1.

**Table 1:** The survey questions, the response options, and the aggregated response numbers

No	Question	Response options	Response number
1	Are you involved in caring for or in the diagnostic process of patients with known or possible rare bone diseases (or conditions where skeletal anomalies and related findings are an important feature)?	I am involved in the diagnostic process	23
		I am involved in the pre- and/or post-diagnosis patient care	6
		I am involved with both: diagnostic process and pre/post diagnosis care	69
		I am NOT involved in diagnostic process or pre/post care. Please explain your interest in this survey	5
2	What is your primary role in or related to healthcare?	Physician	92
		Physician Assistant or Nurse Practitioner	1
		Genetic Counselor	6
		Nurse	1
		Researcher (but not a formal clinician)	3
		Other (please specify)	0
3	Which title best describes your position (please check if you have a dual function)?	Endocrinologist	21
		Medical Geneticist	38
		Neurosurgeon	0
		Orthopaedic surgeon	4
		Pediatrician	26
		Radiologist	8
		Internist	0
		Family medicine physician	0
		Other, please specify	11 (See Fig. 2)
4	Are you involved in teaching or training other healthcare professionals?	Yes	96
		No	7
5	How many years of experience do you have in the healthcare field?	Less than 1 year	0
		1–5 years	6
		5–10 years	13
		More than 10 years	84
6	Which type of healthcare facility best describes where you primarily work (i.e., where do you work most often)?	Academic medical center	84
		Community hospital or clinic	8
		Private practice	2
		Research institution	4
		Government medical center	3
		Other (Please specify)	2
7	Please enter the country where you primarily work	(A text field was provided for the response)	See Supplementary Fig. 2
8	What age group(s) of the patients do you work with (check all that apply)?	Neonates and infants (0–1 year old)	86
		1–10 years old	89
		10–18 years old	95
		Greater than 18 years old	63
9	Approximately, how many patients with known or suspected rare bone diseases (or conditions where skeletal anomalies and related findings are an important feature) does your entire facility see per month?	Less than 5 per month	8
		5–10 per month	9
		10–20 per month	19
		20–50 per month	25
		50–100 per month	20
		100–200 per month	11
		Greater than 200 per month	10
10	According to the 2023 revision of the nosology of genetic skeletal disorders (Unger et al.), there are 41 different groups of skeletal disorders. Please SELECT ALL of the groups that represent the patients for which you, your clinic, and/or your institution provide care	The group names and IDs according to Unger et al. (2023) were provided as response options	See Supplementary Fig. 4
11	In your opinion, how important are medical images (i.e., x-rays, MRI, etc.) in the diagnosis of rare bone diseases?	Not at all important	1
		Slightly important	0
		Moderately important	8
		Very important	40
		Extremely important	54
12	Which imaging type do you think is the most important modality for the postnatal diagnosis of rare bone diseases?	Projectional radiography (X-rays)	84
		Computed tomography (CT) scans	8
		Dual-energy X-ray absorptiometry	2
		Magnetic resonance imaging (MRI)	7
13	How difficult do you think it is to delineate between different rare bone diseases based on visual inspection of patients’ radiographs? (for answering this question, you may exclude the disorders with highly characteristic features such as achondroplasia)	Extremely difficult	27
		Somewhat difficult	59
		Neither easy nor difficult	10
		Somewhat easy	6
		Extremely easy	0
14	What regulatory considerations or ethical concerns do you foresee in implementing image recognition AI for rare bone disease diagnosis? Select all that apply	Data privacy and security	64
		Interpretability of AI algorithms	69
		Compliance with medical regulations	53
		Informed consent for AI-based diagnostics	47
15	How concerned are you about the potential for AI-related errors in the diagnosis of rare bone diseases?	Very concerned	14
		Somewhat concerned	31
		Neutral	20
		Not very concerned	25
		Not concerned at all	1
		Unsure, need to see research results first	12
16	As long as the image recognition AI algorithms are confirmed (through trial studies) to perform their tasks accurately, how important to you is the explainability of these algorithms?	Not at all important	3
		Slightly important	16
		Moderately important	33
		Very important	42
		Extremely important	9
17	Would you be willing to do additional training to learn to utilize an image recognition AI tool for rare bone disease diagnosis?	Yes	93
		No	2
		Unsure	8
18	If an image recognition AI is developed that provides you with a prioritized list of syndromes based on a radiograph, how likely are you to consider integrating it into your current diagnostic workflow?	Extremely unlikely	4
		Somewhat unlikely	7
		Neither likely nor unlikely	8
		Somewhat likely	39
		Extremely likely	45

### Participant recruitment and data collection

Survey data were collected from March to September 2024. To identify participants, we obtained names of clinicians associated with current RBD clinics (identified through our network and through web-based searches) as well as through relevant publications. Participants were recruited directly via email (n = 370) by providing a link to a survey from Qualtrics (Provo, Utah, United States) or indirectly via a flyer at scientific talks and conferences (such as the Annual Clinical Genetics Meeting, European Society of Human Genetics Conference, and International Skeletal Dysplasia Society Meeting). The survey link was also distributed via the newsletters of the European Reference Network on Rare Bone Diseases (ERN-BOND, with 50 HCPs as of this writing) and the European Reference Network for Rare Malformation Syndromes, Intellectual and Other Neurodevelopmental Disorders (ERN-ITHACA, with 71 HCPs as of this writing) in March and April 2024, respectively. The only inclusion criteria was involvement in rare bone disease patient care, which was asked by the first question and could also be assessed with follow-up questions. While an open link was provided, the research team verified no duplicate entries were included in the analysis. The survey response rate was calculated by the number of surveys completed divided by the number of participants directly contacted. We followed the Consensus-Based Checklist for Reporting of Survey Studies (CROSS) [13].

## Results

Of the 125 participants who began the survey (provided demographic information), 103 completed the survey and provided information about their clinical experience with RBDs (27.8% response rate). The following results are based on these 103 responses. Table 1 provides the aggregated result to the survey questions and interactive visualizations of the results are also provided on the Bone2Gene website.

### Demography

**Fig. 1:**
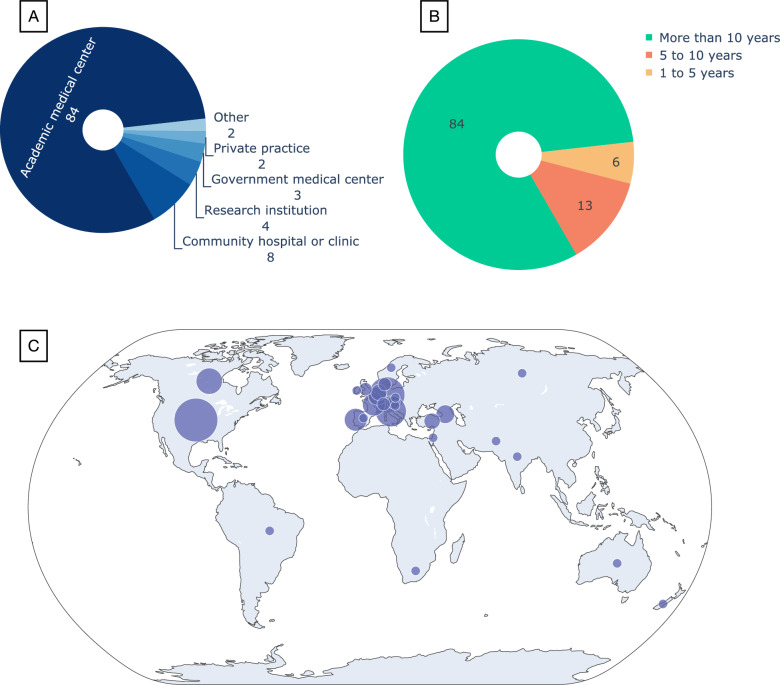
Demographics of the 103 participants who completed the survey. **A** Type of healthcare facility best describing where they primarily work. **B** Years of experience in the healthcare field. **C** The distribution of participants on the world map; the circle sizes reflect the number of participants (see the Supplementary information for the exact number per each of the 27 countries)

Out of a total of 103 complete survey responses 23 individuals (22%) describe themselves as involved in the diagnostic process, 6 (6%) in pre- and post-diagnostic care, and 69 (67%) are involved in both. The remaining 5 (5%) are only involved in research activities in this field. Ninety-two (89%) of the participants are physicians. The majority of participants (84, 81%) primarily work at academic medical centers (Fig. 1A) and have more than 10 years of experience (also 84, 81%, Fig. 1B). Ninety-six (93%) of the respondents are involved in teaching or training other healthcare professionals (Table 1 and Supplementary Fig. 1).

Survey participants represented most global regions (Fig. 1C) and a total of 27 different countries (Supplementary Fig. 2). Fifty-eight (56%) of the respondents are located in Europe and 30 (29%) are in the United States and Canada.

**Fig. 2:**
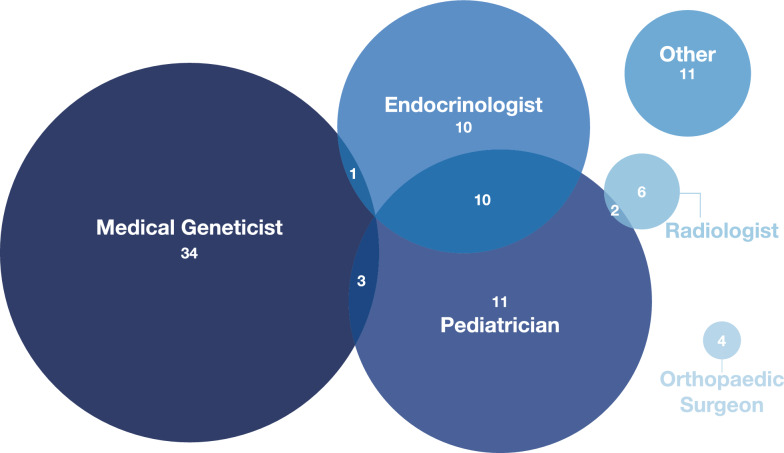
Specialties of the physician participants (n = 92). The most common specialty represented amongst participants was medical genetics. Some participants selected multiple specialties (e.g., endocrinologists who also practice pediatrics, n = 10). Other specialties represented include nephrologist, internist, rheumatologist, pediatric physiatrist, maternal–fetal medicine, general surgeon, pathologist, and geriatrician. We note that countries may have variable requirements regarding initial (general) training versus allowing clinicians to enter a subspecialty immediately, and some clinicians may have responded regarding their current practice rather than their entire training

The diagram in Fig. 2 shows the distribution of the responses from the 92 physician participants to the question “Which title best describes your position?”. Participants could indicate multiple specialties. The most frequent groups were medical geneticists, pediatricians, and endocrinologists, together accounting for 71 (69%) of the respondents.

### Patients statistics

In this section, we report the responses to the questions related to the RBD patients seen by the participants. Fifty respondents (48%) see patients from all four age groups described in our questionnaire, namely 0–1,1–10,10–18, and greater than 18 years old. Further, most of the respondents see patients below 18 years old but 63 (61%) of them also see patients 18 years old or above (Table 1 and Supplementary Fig. 3).

The number of patients with known or suspected rare bone diseases seen by the facilities where the respondents work follows a roughly bell-shaped distribution (Supplementary Fig. 3). Sixty-four (62%) of the facilities see 10–100, seventeen (16%) see less than 10, and twenty-one (20%) see more than 100 patients per month.

According to the most recent revision of the nosology of RBDs [1] there are 41 different groups of skeletal disorders. In one of the questions in our survey, we asked the participants to mark all the groups that they or their healthcare facility have dealt with. The results are visualized in Supplementary Fig. 4. The most frequently observed disorder was the Osteogenesis imperfecta and bone fragility group, with 89% (n = 92) of participants reporting care of patients with this group of conditions at their institution. This was followed by Disorders of bone mineralization (n = 89, 86%) and *FGFR3* chondrodysplasias (n = 88, 85%).

### Role of imaging in diagnosing RBDs

Ninety-four (91%) of the respondents find imaging to be very or extremely important, and the majority (n = 84, 81%) consider X-rays to be the most important imaging modality (Table 1 and Supplementary Fig. 5).

Further, our result (Table 1 and Supplementary Fig. 6) shows that most participants, 83% (n = 86) regardless of years of experience, find the interpretation of patients’ radiographs and delineation of different RBDs based on visual inspection to be somewhat (n = 59) or extremely difficult (n = 27).

### Participants’ perception of the use of AI

**Fig. 3:**
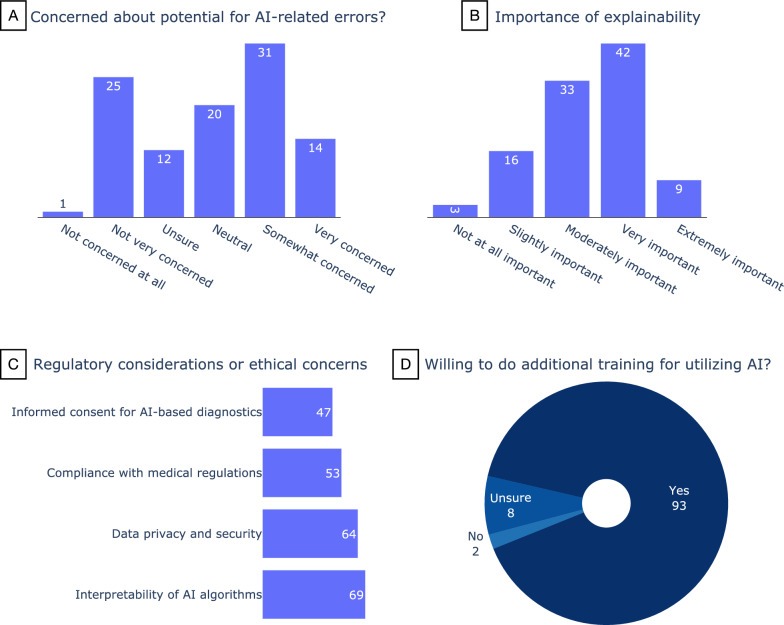
Responses of the participants regarding **A** concerns about potential AI errors, **B** importance of explainability, **C** regulatory considerations, and **D** willingness to undergo further training for using AI

Forty-four percent of the participants (45/103) are somewhat (n = 31) or very (n = 14) concerned about AI-related errors, while 24% (n = 25) are not very concerned (Fig. 3A). Around half of the participants (n = 51) consider the explainability of AI algorithms to be very (42/103) or extremely (9/103) important (Fig. 3B). There was no relation between the years of experience of the participants and their answers to these questions.

**Fig. 4:**
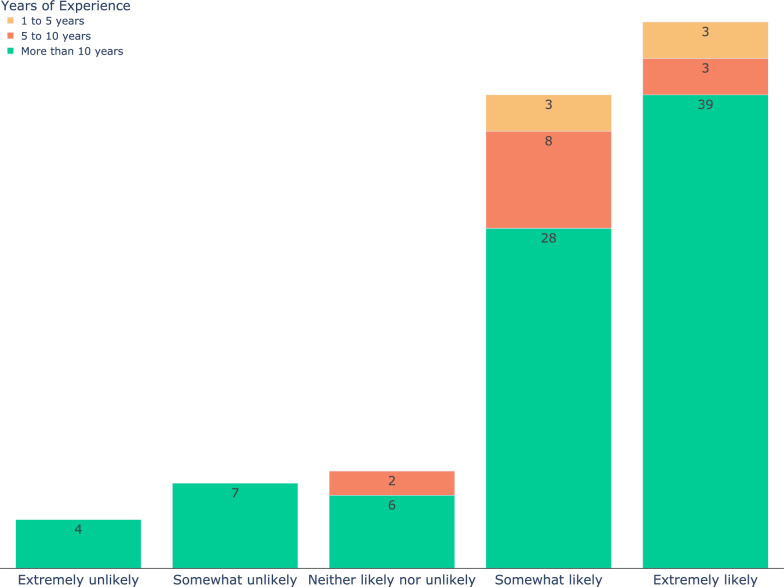
The responses of the participants to the question “If an image recognition AI is developed that provides you with a prioritized list of syndromes based on a radiograph, how likely are you to consider integrating it into your current diagnostic workflow?”. The color code shows the years of experience. Eighty-one percent of the participants (including all those with 1–5 years of experience) responded somewhat (n = 39) or extremely (n = 45) likely

On the subject of regulatory considerations or ethical concerns (Fig. 3C), the interpretability of AI algorithms received the most attention (n = 69) followed by data privacy and security (n = 64). Ninety-three (90%) of the participants indicated they would be willing to do additional training for utilizing AI (Fig. 3D), and when asked how likely they are to consider integrating image recognition AI into their current diagnostic workflow, 81% responded they were somewhat (n = 39) or extremely (n = 45) likely (Fig. 4). Forty-five participants expressed interest in further collaborations in this area.

## Discussion

To address the need for a multidisciplinary approach [14, 15], it is crucial to gather insights from a wide range of specialties involved in the diagnosis and management of RBDs. We primarily targeted clinicians for participation, which would encompass most but not all persons involved in clinical care. Further, while most of the participants in our survey were medical geneticists, pediatricians, and endocrinologists, our survey also managed to reach radiologists, orthopedic surgeons, nephrologists, internists, rheumatologists, pediatric physiatrists, maternal–fetal medicine, general surgeons, pathologists, and geriatricians. However, a limitation of our study is the underrepresentation of the latter specialist groups, likely due to our recruitment strategy, which primarily targeted geneticists and endocrinologists. In particular, it is very important to gauge the opinion of radiologists on the use of AI tools for analyzing radiographs. Multiple studies in the literature have addressed this topic [16–19] reporting a generally positive attitude of radiologists towards AI. However, these studies have gauged the general expectations of radiologists and were not focused on RBDs. Future survey studies should try to reach more radiologists who are active in the field of RBDs. 

International collaboration plays a crucial role in advancing research and development for rare diseases [20]. Our survey managed to gather data from participants from 27 countries around the world. The majority of the participants (n = 88, 85%) are located in Europe, United States, and Canada. However, despite trying to share the survey link widely via our network, we unfortunately received no responses from East and Southern Asia. Further, we received very few responses from West Asia (Middle East), Africa, Australia, and South America, hence, our sample is not representative of HCPs in those regions. This highlights the need for larger efforts to expand cross-regional connections and collaborations, including addressing issues such as language barriers (our survey was only offered in English) and different medical system practices [21].

Despite advances in new sequencing technologies and efforts at variant classification, many sequencing findings remain variants of uncertain significance (VUS), interpretations of which may require phenotypic examinations by trained dysmorphologists [22], and establishing correlations between genotype and phenotype (e.g., radiologic findings) can be essential for the final diagnosis [23]. Our results indicate that the majority of respondents find imaging (and in particular radiographs) to be highly important.

The opportunities and challenges of using AI in medicine and healthcare, in particular ethical concerns, privacy issues, and interpretability and explainability of AI models, have been discussed in various studies [22, 24–26]. Respondents to our survey echoed these concerns, although they generally expressed a positive attitude toward the use of AI.

Similar to the results from [27] reporting interviews with 20 different stakeholders about the adoption of facial recognition algorithms for rare disease diagnosis, the majority of the participants of our survey are also very likely to consider integrating an image recognition AI into their current RBD diagnostic workflow and to undergo further training for using AI. 

## Conclusion

In this study, we conducted a survey to gather feedback from potential users of an AI-based diagnostic tool. The aim was to integrate their input into the development process and reduce the information asymmetry between AI researchers and clinicians, who possess essential insights into the practical challenges and needs of diagnosing RBDs. Most survey respondents indicated they would be willing to incorporate AI into their diagnostic workflow for RBDs. However, AI-related errors, privacy issues, and interpretability and explainability of AI models remain the most important concerns of healthcare professionals regarding AI adoption. This highlights the need for close and transparent collaboration between developers and healthcare professionals before and during the design, testing, and implementation of AI-based tools. 

## Supplementary Information

Additional file1 (PDF 255 KB)

## Data Availability

The datasets generated during the current study are not publicly available due preserving the privacy of individual survey participants, but the aggregated results used for analysis and producing the figures are provided in Table 1 of the manuscript and are also provided as a supplementary Excel sheet.
