# Supplementary material for: Artificial intelligence for diagnosing rare bone diseases: a global survey of healthcare professionals
Source: Orphanet J Rare Dis. 2025 Jul 16;20:365. doi: 10.1186/s13023-025-03875-1 (PMC12265221; doi:10.1186/s13023-025-03875-1)
Supplement: Supplementary file 1 — Additional file1 (PDF 255 KB) [file 13023_2025_3875_MOESM1_ESM.pdf]

Supplementary information for the manuscript

Artificial Intelligence for Diagnosing Rare Bone Diseases: A Global Survey of Healthcare Professionals

Behnam Javanmardi[*,§,1], Rebekah L. Waikel[*,2], Tinatin Tkemaladze[3,4], Shahida Moosa[5], Alexander Küsshauer[6], Jean Tori Pantel[7], Minu Fardipour[8], Peter Krawitz[1], Benjamin D. Solomon[2], Klaus Mohnike[8]

* Equal contribution
§ Corresponding Author

1- Institute for Genomic Statistics and Bioinformatics, University Hospital Bonn, Bonn, Germany
2- National Human Genome Research Institute, Maryland, United States
3- Department of Molecular and Medical Genetics, Tbilisi State Medical University, Tbilisi, Georgia
4- Division of Clinical Genetics, Givi Zhvania Pediatric University Clinic, Tbilisi State Medical University, Tbilisi, Georgia
5- Stellenbosch University, Stellenbosch, South Africa
6- Transfer Center enaCom, University of Bonn, Bonn, Germany
7- Institute for Digitalization and General Medicine, Medical Faculty, RWTH Aachen University, Aachen, Germany
8- University Hospital Magdeburg, Magdeburg, Germany

We present the supplementary figures of our survey study results here. The complete survey and results with interactive visualizations can also be accessed on the Bone2Gene website (https://bone2gene.org).

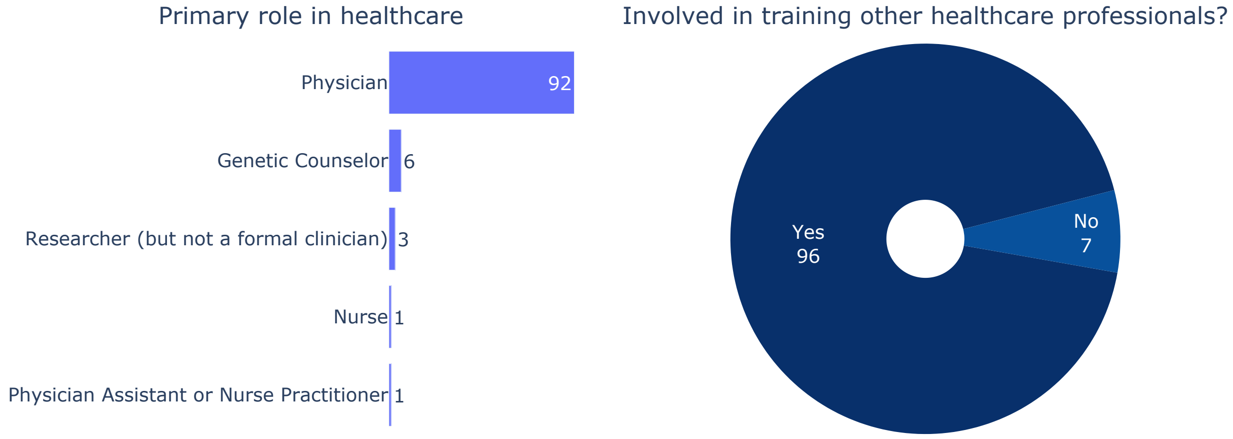

Supplementary Figure 1: Professional demographics of the 103 participants who completed the survey. Primary role in or related to healthcare (left). Involvement in teaching or training other healthcare professionals (right).

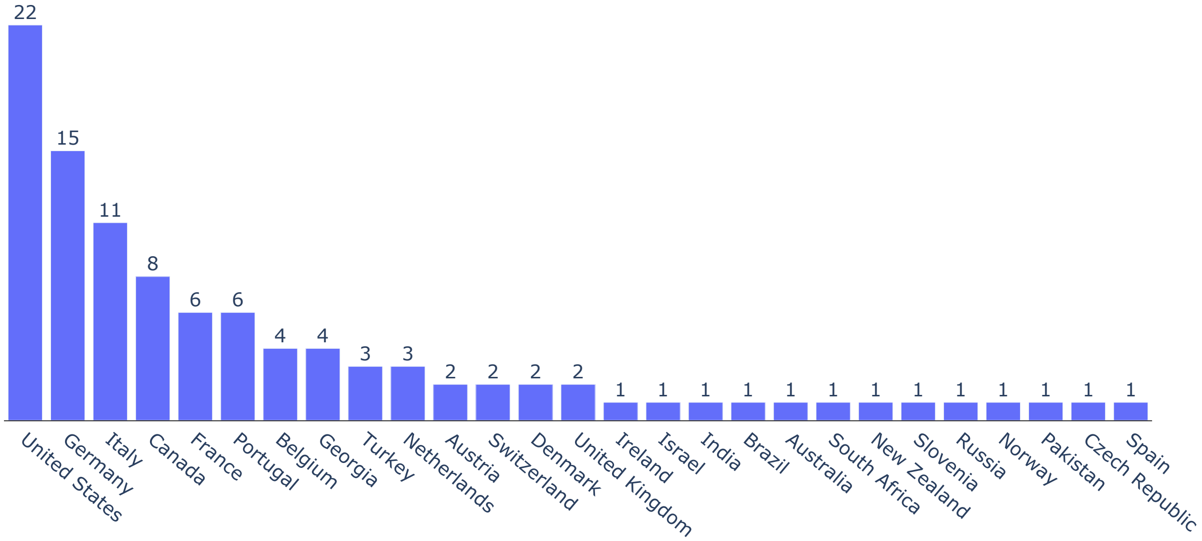

Supplementary Figure 2: The number of participants per each of the 27 different countries.

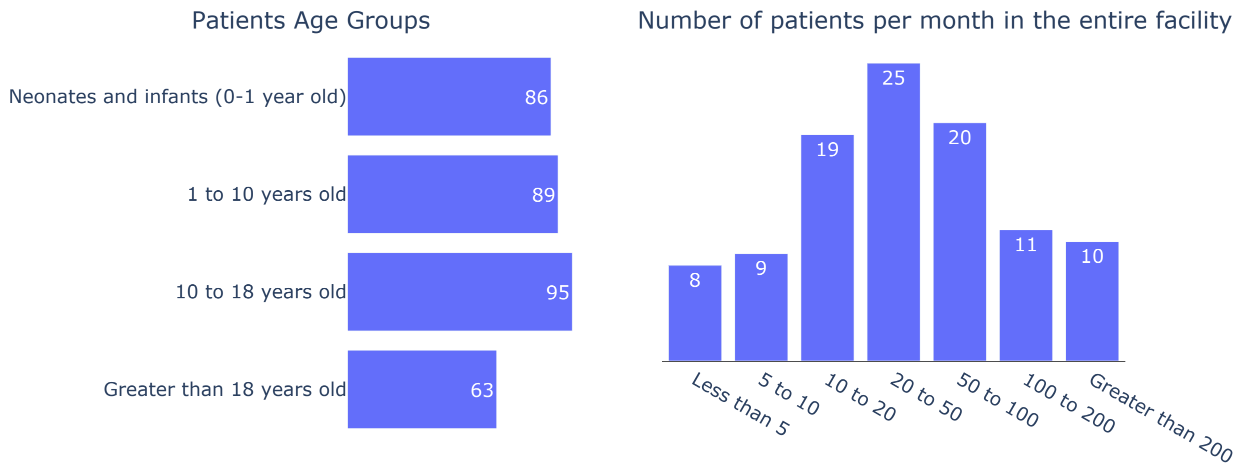

Supplementary Figure 3: Left: The age group(s) of the patients that the respondents work with (multiple options could be selected). Right: The number of patients (per month) with known or suspected RBDs (or conditions where skeletal anomalies and related findings are an important feature) for whom the entire facility of the participant provides care.

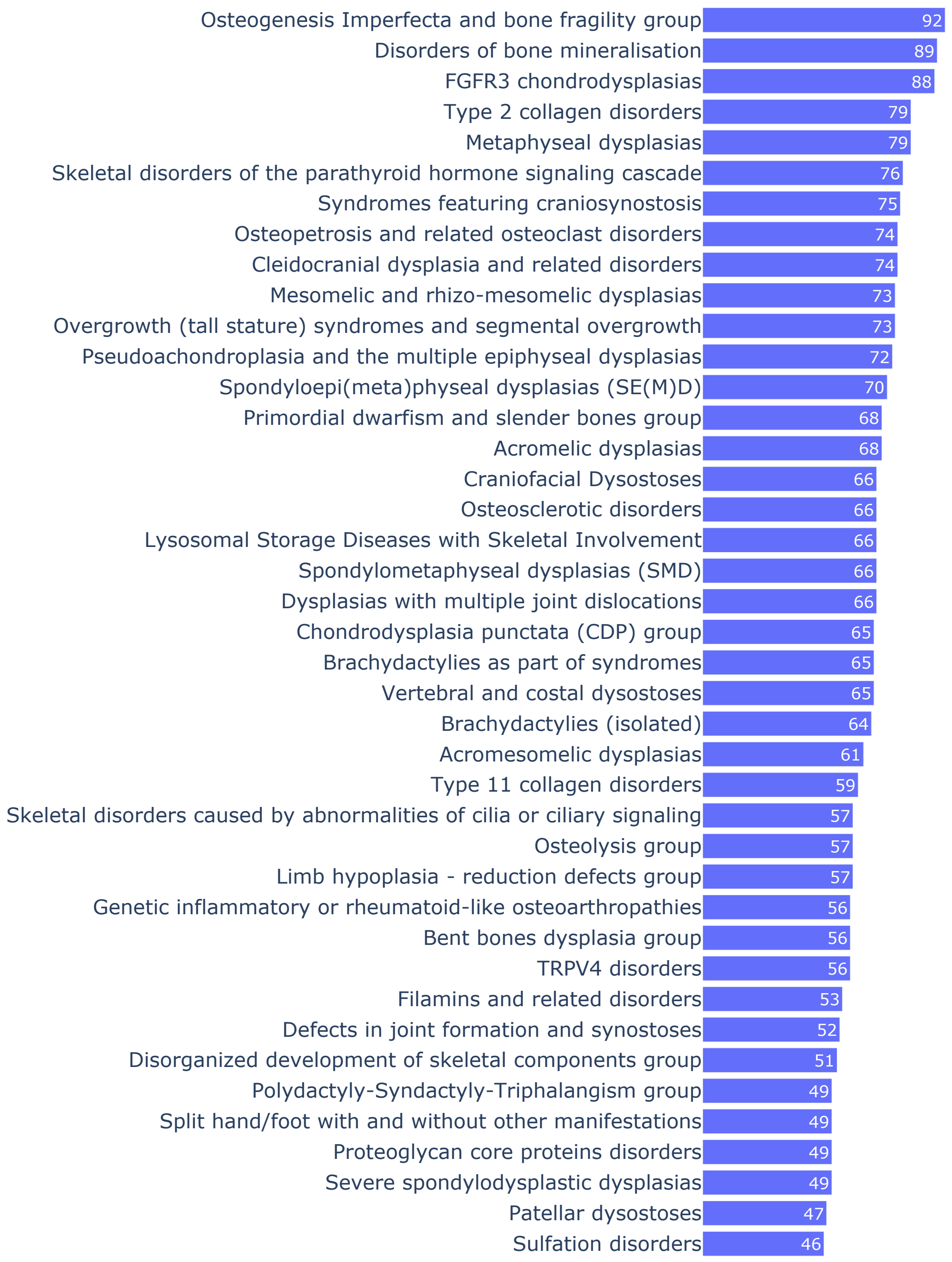

Supplementary Figure 4: Most frequently observed skeletal disorders. According to the 2023 revision of the nosology of RBDs (Unger et al.) there are 41 different groups of skeletal disorders. Participants reported the groups representing the patients for which they, their clinic, and/or their institution provide care (100 out of 103 survey respondents answered this question). The most frequently observed disorder was Osteogenesis Imperfecta and bone fragility group with 89% (92/103) of participants reporting care of patients with this condition at their institution.

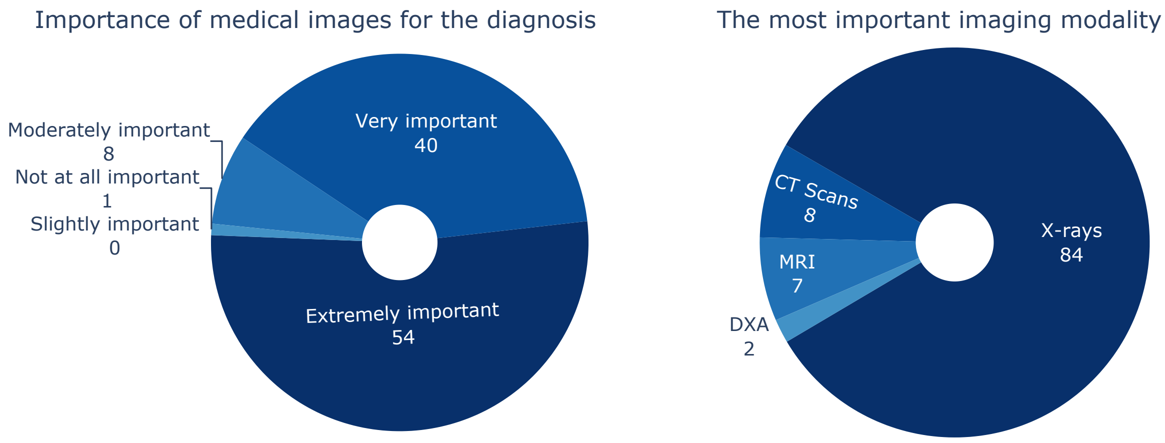

Supplementary Figure 5: Left: Importance of medical imaging for RBD diagnosis. Most participants reported imaging to be very important or extremely important in the diagnosis of RBDs (n=95). Right: The most important imaging modality. Most of the participants reported X-rays to be their preferred imaging modality for diagnostic images (n=85).

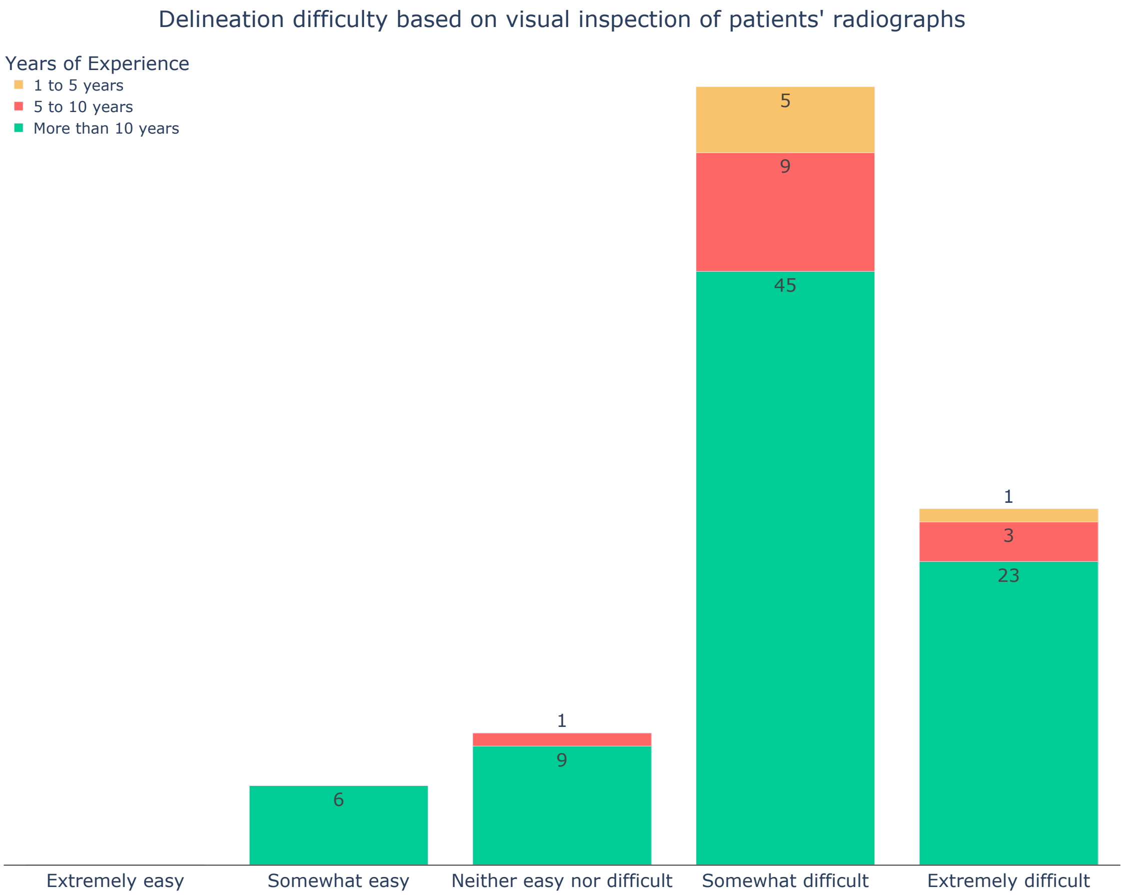

Supplementary Figure 6: The distribution of responses to the question “How difficult do you think it is to delineate between different rare bone diseases based on visual inspection of patients' radiographs? (for answering this question you may exclude the disorders with highly characteristic features such as achondroplasia)”. We color-coded the responses based on the participants' years of experience. Most respondents, 83% (n=86) regardless of years of experience, reported diagnostic interpretation and delineation of different RBDs based on visual inspection of diagnostic imaging to be somewhat (n=59) or extremely difficult (n=27). The majority of those with less than 10 years of experience, 95% (18/19), reported that this was somewhat to extremely difficult compared to 82% (68/83) with at least 10 years of experience.
